# Molecular evolution and signatures of selective pressures on *Bos*, focusing on the Nelore breed (*Bos indicus*)

**DOI:** 10.1371/journal.pone.0279091

**Published:** 2022-12-22

**Authors:** Thainá Cortez, Horácio Montenegro, Luiz L. Coutinho, Luciana C. A. Regitano, Sónia C. S. Andrade

**Affiliations:** 1 Departamento de Genética e Biologia Evolutiva, Universidade de São Paulo (USP), São Paulo, SP, Brazil; 2 Departamento de Zootecnia, Escola Superior de Agricultura Luiz de Queiroz, Universidade de São Paulo (ESALQ), Piracicaba, SP, Brazil; 3 Empresa Brasileira de Pesquisa Agropecuária, Embrapa Pecuária Sudeste, São Carlos, SP, Brazil; University of Bologna, ITALY

## Abstract

Evolutionary history leads to genome changes over time, especially for species that have experienced intense selective pressures over a short period. Here, we investigated the genomic evolution of *Bos* species by searching for potential selection signatures, focusing on Nelore, an economically relevant cattle breed in Brazil. We assessed the genomic processes determining the molecular evolution across Nelore and thirteen other related taxa by evaluating (i) amino acid sequence conservation, (ii) the dN/dS ratio, and (iii) gene families’ turnover rate (λ). Low conserved regions potentially associated with fatty acid metabolism seem to reflect differences in meat fat content in taxa with different evolutionary histories. All *Bos* species presented genes under positive selection, especially *B*. *indicus* and Nelore, which include transport protein cobalamin, glycolipid metabolism, and hormone signaling. These findings could be explained by constant selective pressures to obtain higher immune resistance and efficient metabolism. The gene contraction rate across the Nelore + *B*. *indicus* branch was almost nine times higher than that in other lineages (λ = 0.01043 *vs*. 0.00121), indicating gene losses during the domestication process. Amino acid biosynthesis, reproductive and innate immune system-related pathways were associated with genes recognized within the most frequent rapidly evolving gene families and in genes under positive selection, supporting the substantial relevance of such traits from a domestication perspective. Our data provide new insights into how the genome may respond to intense artificial selection in distinct taxa, and reinforces the presence of selective pressures on traits potentially relevant for future animal breeding investments.

## Introduction

Domesticated species, such as sheep, horses, pigs, goats, and cattle, have been subjected to long periods of intense phenotypic selection that have resulted in dramatic genomic changes in a few generations [1]. Modern cattle, the most important domesticated group, with more than 900 million heads across the globe, can be grouped into humpless taurines (*Bos taurus*) and humped zebuines (*Bos indicus*). These species differ in many morphological and physiological aspects, but most commonly by the prominent hump and long face in the zebu. In Brazil, the humped cattle Nelore constitutes the largest part of the commercial herd, mainly because of its advantages in tropical environments, such as greater thermal tolerance, lower metabolic rate and nutrient requirement, natural resistance to ectoparasites, and higher fertility [2–4]. Previous studies [5, 6] indicated that zebu and taurine cattle lineages share the ancestor aurochs, *Bos primigenius*, and that the humped cattle evolution presents multiple domestication historical scenarios, where the African lineage not only seem to contain the oldest zebu lineage, but also exhibit a very particular evolutionary narrative [7].

Domestication includes artificial selection, but also the relaxation of selection pressures on several other predictors (e.g., hunting, predation, and starvation). The effects of selective breeding on economically relevant traits have been investigated by identifying footprints across the entire genomic architecture, such as high allele frequencies, increased amino acid change rates, and low genetic diversity [1, 8]. In this context, Somavilla *et al*. [9] studied selection signatures in Nelore cattle, which revealed several regions related to reproduction, growth, meat quality, and fatty acid content under recent selection. Evolutionary approaches have demonstrated high efficacy for the study of genomic signatures resulting from domestication, especially because recently developed methods have progressively improved accuracy. Phylogenetic analysis of homologous sequences, for example, evaluates the amount of divergence between two or more species by quantifying the substitution rate [e.g., 10–13]. Gene family’s evolution rate, based on the size of gene copies, allows us to not only infer the probability of the observed changes resulting from natural selection, but also to identify genomic changes into expansion and/or contraction rates [14–16]. Although rapid gene family expansion may indicate positive selection, the pressures underlying gene family contraction/loss are less clear and could be produced by both neutral and adaptive regimes. Despite being a useful tool to complement and benefit evolutionary studies, gene family evolution investigations remain understudied when compared to the analysis of homologous sequences [17].

In the present study, we aimed to evaluate the evolutionary history and potential signatures of selection in Nelore (*B*. *indicus*) genomic regions, as well as in four other *Bos* species, including *B*. *taurus*. For this purpose, we used thirteen related taxa to assess the molecular evolution rate by evaluating (i) the similarity score of amino acid sequences across all taxa and between Nelore and *B*. *taurus*, separately, (ii) the dN/dS ratio variation across all taxa, and (iii) the gene family turnover rate across the lineages of interest. We expected to find signs of positive selection in taxa that have experienced intense domestication for long periods, focusing on Nelore. Finally, we focused on genomic regions under selection potentially associated with economically relevant functional categories from a domestication perspective.

## Materials and methods

### Dataset achievement and orthologous groups assignment

All procedures related to animal experiments were undertaken following the Institutional Animal Care and Use Committee Guidelines (IACUC) from the Brazilian Agriculture Research Corporation (EMBRAPA) and approved by the director Dr. Rui Machado. To assemble the Nelore transcriptome, samples of the *longissimus dorsi* muscle from 43 bulls in the population were collected for RNA sequencing using the HiSeq 2000 Illumina platform (San Diego, CA). All details about the birth, growth, animals’ selection criteria and experiments are presented in Cesar *et al*. [18]. The RNA extraction protocol, quality analysis, library preparation, and sequencing platform description are detailed in Poleti *et al*. [19]. This transcriptome was obtained with the specific purpose of evaluating the regulatory factors associated with fat content of Nelores’ muscle, so for this reason we chose to investigate which potential genes were encompassed in the breed. The raw reads from the 43 bulls used in this study are publicly available in the European Nucleotide Archive (ENA) repository (EMBL-EBI), under accession PRJEB13188. Approximately 350 million obtained reads were filtered using the Seqyclean v. 1.9 [20] with a Phred-Score cut-off of 26, a minimum size of 65bp, and contaminants identified from UNIVec (https://www.ncbi.nlm.nih.gov/tools/vecscreen/univec/). After filtering, approximately 284 million reads were normalized using the normalize_by_kmer_coverage.pl script from Trinity v. 2.1.1 [21], leaving 133,384,146 reads. The *de novo assembly* was also performed using Trinity, where only contigs longer than 300 bp were retained. CD-HIT v. 4.6 [22] removed redundant contigs, using a limit of 95% overall similarity. The diversity and completeness of the transcriptomes were assessed by predicting the orthologs using 3,354 conserved vertebrate genes (ODB10) with Benchmarking Universal Single-Copy Orthologs (BUSCO) v.5.0 [23]. The TransDecoder v. 2.0.1 [24] identified open reading frames and translated the coding sequences (CDs). Annotation was performed using the BLASTX tool [25] using the UniProt database with an e-value = 1e-5 as cut-off.

Nucleotide sequences from the nr database and amino acid data were downloaded from the NCBI for 13 related species, chosen according to the data quality and availability in GenBank: *Bison bison*, *Bos grunniens*, *Bos indicus*, *Bos mutus*, *Bos taurus*, *Bubalus bubalis*, *Capra hircus*, *Equus caballus*, *Equus przewalskii*, *Muntiacus muntjak*, *Ovis aries*, *Pantholops hodgsonii*, and *Sus scrofa* (Table 1).

**Table 1:** The thirteen taxa chosen for this study and respective data.

Taxa	Amino acid sequences	Nucleotide sequences	Domesticated
*Bison bison*	35,698	189,258	No
*Bos grunniens*	1,953	1,777	Yes
*Bos indicus*	2,277	52,374	Yes
*Bos mutus*	47,595	112,226	No
*Bos taurus*	127,289	231,900	Yes
*Bubalus bubalis*	45,817	1,030,307	Yes
*Capra hircus*	48,559	88,639	Yes
*Muntiacus muntjak*	139	361	No
*Ovis aries*	106,666	112,681	Yes
*Pantholops hodgsonii*	32,338	60,339	No
*Sus scrofa*	69,780	548,285	Yes
*Equus caballus*	50,961	146,304	Yes
*Equus przewalskii*	38,809	109,141	No

The number of amino acid/nucleotide sequences available at NCBI and the domestication status are shown for each taxon.

Both amino acid and nucleotide datasets were separately assigned to orthologous groups (OGs) using the OrthoMCL v. 2.0.9 [26]. The MCL inflation parameter was varied in increments of 0.2, ranging from 1.6 to 2.4. The final cluster composition was not particularly sensitive to different inflation values in this range. An inflation value of 2.0 was used, which is within the range of inflation parameters used in similar studies. All clusters were aligned using MAFFT v. 7.271 [27]. To identify putative OGs, we used the RAxML v. 7.2 [28] with 1,000 bootstraps to construct a phylogenetic tree for each dataset. Monophyly masking was conducted to reduce the number of monophyletic sequences from the same taxon to one sequence by an iterative paralogy pruning procedure using PhyloTreePruner (http://sourceforge.net/projects/phylotreepruner/) [29]. The inclusive subtrees with no more than one sequence per taxon were pruned and retained. Files generated from subtrees were aligned using MAFFTL-INS-i v. 7.271.

**Fig 1:**
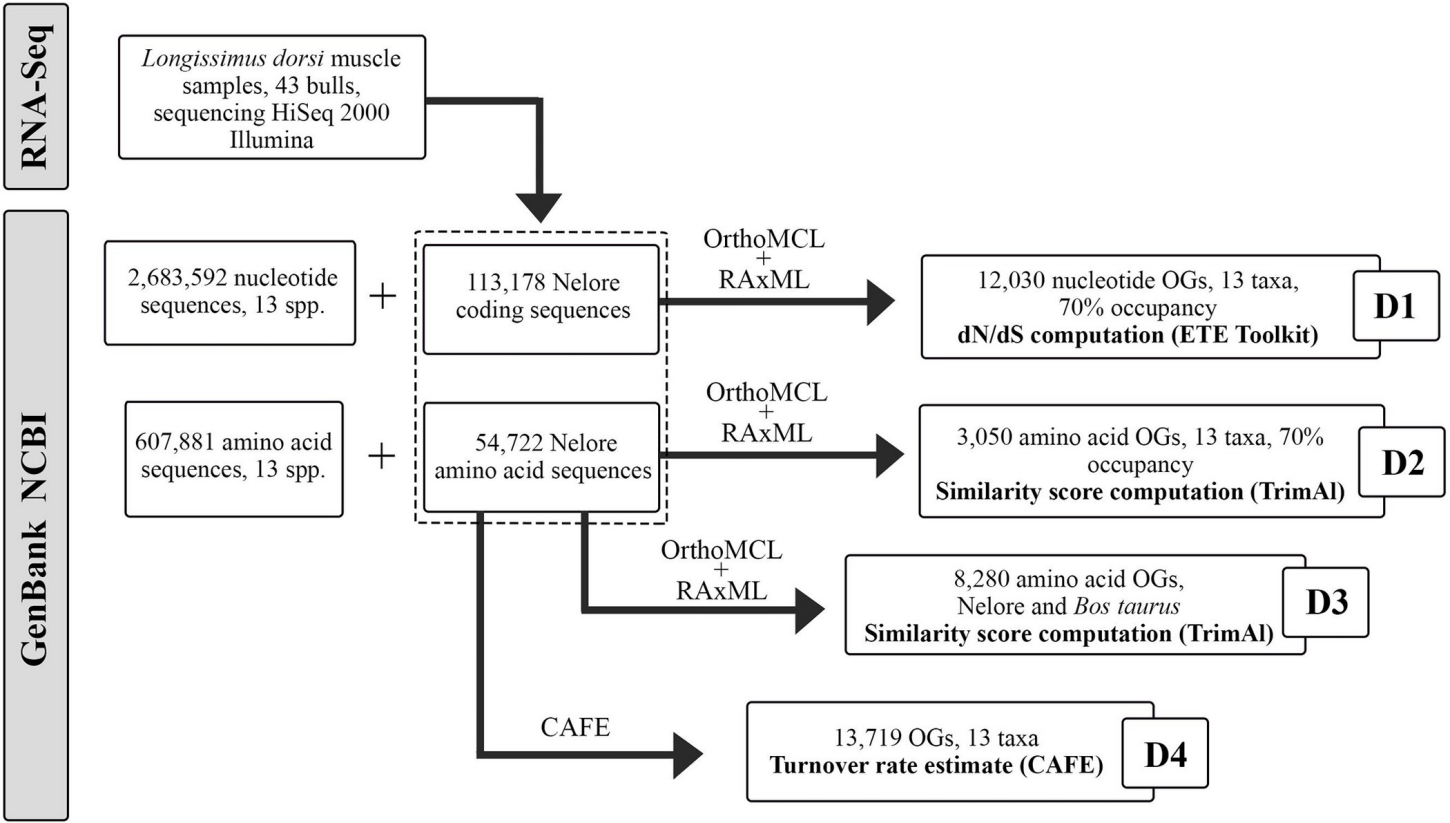
Schematic description of each dataset, orthologous groups (OGs) assignment and analyses performed.

By applying command line scripts, only OGs containing at least 10 taxa were retained (~75% occupancy). Furthermore, amino acid OGs with both Nelore and *B*. *taurus* data were subsampled into a new dataset. Therefore, three datasets were constructed: nucleotide OGs from 13 taxa (hereafter D1), amino acid OGs from 13 taxa (D2), and amino acid OGs containing only Nelore + *B*. *taurus* sequences (D3) (Fig 1).

### Similarity score

To measure the conservation level among amino acid sequences, we used the program TrimAl v.1.2 [30]. The program reads all sites of the alignment and then computes the residue similarity score (*S*_*S*_), which consists of mean distance (MD) scores of all sequences contained in each OG. Based on the MD scores, we calculated the average similarity value of each cluster. The sequences conservation level was estimated for the all the 13 taxa together (D2) and for Nelore + *B*. *taurus* (D3).

### Selection test

We first performed a selection analysis on the nucleotide OGs (D1, Fig 1) with ETE Toolkit v. 2.3 [31] under different evolutionary models. The program uses the number of synonymous (dS) and non-synonymous changes (dN) to infer the omega value (dN/dS = ω) for a single site, codon, genome, or population. While ω < 1 indicates a signal of purifying selection, ω > 1 indicates a positive selection signal, and ω = 1 indicates neutral evolution [32]. First, we performed the site model comparing the neutral model (M1) to the positive selection model (M2). Model M1 compares all species sequences with each other, and Model M2 compares all species using Nelore as the reference taxa. Non-significant differences between ω[M1] and ω[M2] log likelihood values indicate neutral evolution; if ω[M1] > ω[M2], purifying selection, and if ω[M1] < ω[M2], positive selection. All the clusters with significant difference between the log likelihood values (*p* < 0.05) between the M1 and M2 model values (ω[M1]—ω[M2], hereafter Δω) were submitted to a second test under the branch-test model [33]. This model allows the ω ratio to vary among branches in the phylogeny. The software compares the average of the foreground ω values (branches of interest, ω_F_) with the average of the background ω values (all other branches, ω_B_). If ω_F_ fits the data better, ω_F_ is tested against a null model (ω_F_ = 1), to infer the selection result. Each run was implemented with one species as the foreground branch. Only the genes that exhibited a signal of positive selection for Nelore were used in this approach to evaluate how the selective pressures vary across taxa that experienced artificial selection for long periods sharing a similar evolutionary history. The rate of OGs under positive selection was assessed separately by dividing the number of clusters with ω > 1 by the total number of OGs in each species.

### Gene family evolution analysis

CAFE v. 5 [34] was used to identify gene families with rapid expansions and contractions in copy numbers in the *Bos* lineage by using the same amino acid sequences downloaded from NCBI. Following the CAFE tutorial, an all-against-all BLASTP [25] was applied, followed by the application of the MCL-14-137 clustering algorithm [35, 36]. The phylogenetic hypothesis was then constructed using only OGs containing the Nelore OTU and with 70% occupancy, a total of 6,850 out of 13,719 OGs (dataset D4, Fig 1). The species tree was generated using the maximum-likelihood approach of IQTree v1.6.12 [37] using the LG4X+G model and 1,000 bootstrap replicates. We estimated the number of gene families across all OGs and the birth and death rates across different lineages, represented by the genomic turnover (λ) per gene family per million years. To identify rapidly changing gene families, CAFE estimates the variance in gene family size. Large variance (cut-off p-value * * =  0.01) indicates expansion or contraction events [38]. CAFE infers the distribution of error (*e*) regarding the number of gene copies present in the dataset, and estimates the turnover rate λ. We compared the global model with one λ rate for all branches to the multi-λ model with different rates of gene turnover between the Nelore + *B*. *indicus* branch and the remaining tree. By doing this, we can infer whether different λ rates better explain the data and, if so, how much these evolution rates differ. The best model was inferred via likelihood ratio computation (2×[*lnLglobal−lnLmulti*]) after CAFE employed a set of simulations for the global model. To assess potential biases in Nelore + *B*. *indicus* branch rate, we also ran the multi-λ model individually for these species. Using the BLASTP tool from BLAST v. 2.11 [25], we obtained the functional annotation for all gene families under rapid expansion or contraction.

### Mapping and enrichment analysis

To identify the most represented functional categories associated with extremely low or high identity among taxa and gene clusters under positive selection, we performed both mapping and functional annotation using the Blast2GO software with default parameters [39], which uses the Fisher’s Exact *Test* corrected for multiple comparisons (FDR < 0.05). To assess the gene functions associated to highly conserved and variable genomic regions across these species, we analyzed the metabolic pathways of OGs presenting similarity scores (i) higher than 0.70 and lower than 0.30, among all taxa, (ii) equal to one and lower than 0.60, between Nelore and *B*. *taurus*, (iii) with dN/dS > 1. The different threshold applied between (i) and (ii) is due to the distinct similarity score distribution found when analyzing all taxa and only Nelore and *B*. *taurus* (see results section). The reference background was constructed based on all the OGs included in each analysis. The overrepresented gene ontology terms (hereafter, GOs) within a set of genes of interest were graphically visualized using REVIGO [40]. We considered categories significantly enriched with a p-value < 0.05, so we would not restrict the number of potentially enriched pathways.

## Results

### Datasets

The NCBI database search yielded 607,881 amino acid sequences and 2,683,592 nucleotide sequences available for the 13 taxa, ranging from 139 (amino acid sequences of *M*. *muntjak*) to 1,030,307 sequences per species (nucleotide sequences of *B*. *bubalis*) (Table 1).

The Nelore *de novo* transcriptome assembly presented 366,987 contigs, with an N50 of 3,166 and a mean contig length of 1,446.3. Approximately 100,277 (~27%) had hits against the UniProt database, and 113,178 contigs were translated by TransDecoder (all details and additional statistics are presented by Poleti *et al*. [19]. BUSCO analysis using conserved vertebrate genes showed that 87.3% of these 3,354 sequences were complete in the assembly, 4.4% were fragmented, and 8.3% were missing. These values indicate that, although the transcriptome was constructed based on a single tissue, the dataset retains a high genomic diversity, minimizing the chances of analyses bias. After ORFs identifications and quality filtering steps, OrthoMCL produced 12,030 nucleotide OGs (D1) and 3,057 amino acid (D2) OGs with 70% occupancy. The dataset comparing *B*. *taurus* to Nelore had 8,275 amino acid OGs (D3). Furthermore, our transcriptomic data showed high completeness according to BUSCO, minimizing the chances of bias due to low genomic representation.

### Gene conservation analysis

The conservation among amino acid sequences was estimated using a similarity score (*S*_*S*_) of all sequences contained in each OG. Based on the distribution of similarity scores of each dataset (D2 and D3), we performed the subsequent analyses for the most and least conserved sequences, chosen accordingly to the preliminary results (S1 Fig). The conservation level analysis for the 13 taxa dataset (D2) showed that more than 60% of the OGs (1,843) presented scores over 0.70 (S1A Fig), supporting high conservation across the analyzed sequences when comparing all taxa. To infer which functions or processes these genes are related to, Gene Ontology enrichment analyses were performed. For the clusters with *S*_*S*_ > 0.70, enrichment analysis showed that 528 GOs significantly overrepresented were RNA metabolic process, nucleic acid metabolic process, and organelle organization (S2A Fig and S1 Table). The enrichment analysis of amino acid OGs with *S*_*S*_ < 0.30, revealed 246 overrepresented GOs (S2B Fig and S1 Table). Some of these pathways are part of routes related to the regulation of insulin secretion, which is involved in the cellular response to glucose stimulus, fatty acid elongation, amino acid transport, and fertilization.

From the 8,280 amino acid OGs from D3 (Nelore and *B*. *taurus*), the similarity analysis revealed no *S*_*S*_ < 0.40, and approximately 40% orthogroups (3,305) exhibiting 100% identity (S1B Fig). Enrichment analysis of the identical amino acid OGs revealed 261 overrepresented GOs, including amino acid metabolic process, cellular response to growth factor stimulus, protein stabilization, cholesterol esterification, and others (S3A Fig, S2 Table). OGs with 0.40 < *S*_*S*_ < 0.60 revealed 229 GO routes significantly overrepresented, such as melanocyte migration, cytokines related, T cell immunity, response to fungicide, and respiratory gaseous exchange (S3B Fig, S2 Table).

### Selection test

**Fig 2:**
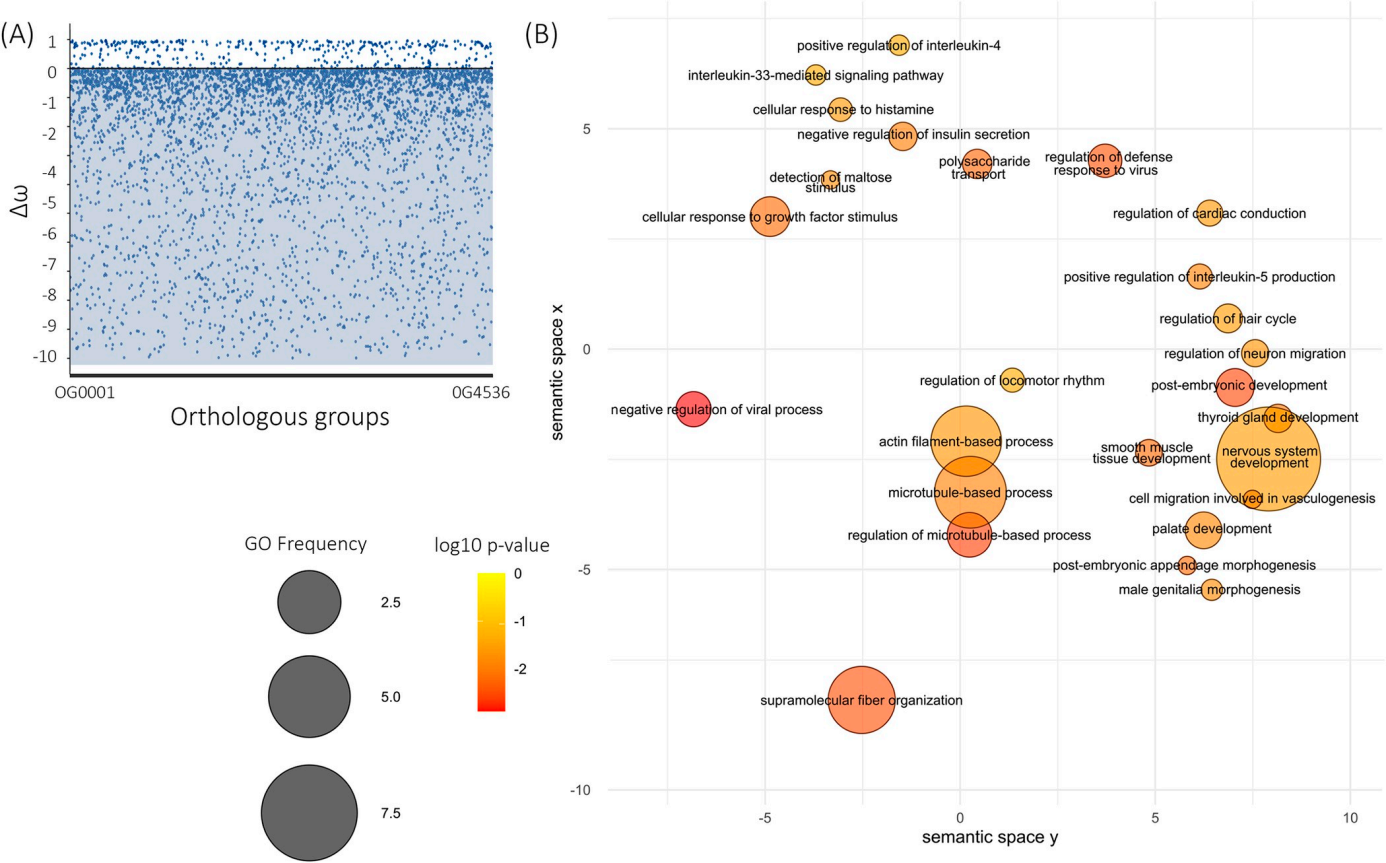
Results of selection test for neutral model (M1) vs. selection model (M2) per OGs and enrichment gene ontology for clusters under positive selection. (a) Δω value per OG, where negative values indicate potential positive selection. Each dot represents one single gene cluster and respective Δω on the y-axis. (b) Enrichment Gene Ontology (GO) result from OGs under positive selection for Nelore. Each circle represents an over-represented GO term: the colors indicate the log10 of the p-value; the size reflects the frequency of the GO.

Molecular evolution analyses were performed to infer the selection effect on candidate sequences or on regions linked to these sequences. The ratio between the number of synonymous (dS) and non-synonymous changes (dN) was calculated to infer the omega value (dN/dS = ω), where ω < 1 indicates negative selection, ω > 1 indicates positive selection, and ω = 1, neutral evolution. The selection test based on the branch model performed with ETE Toolkit over the 12,030 nucleotide OGs (D1) revealed 4,536 orthogroups (~37%) with statistically significant differences between the ω[M1] and ω[M2] values (*p* < 0.05, S3 Table), where 4,115 (~90%) presented negative Δω, indicating a positive selection signal (Fig 2A). From these, 655 exhibited Δω > 10 and were therefore removed due to potential alignment biases. Approximately 2,500 out of the 3,460 OGs under positive selection were annotated. The enrichment analysis revealed 610 overrepresented GOs, including steroid hormone-mediated signaling pathway, stress fiber assembly, and regulation of actin filament bundles (Fig 2B, S4 Table).

**Fig 3:**
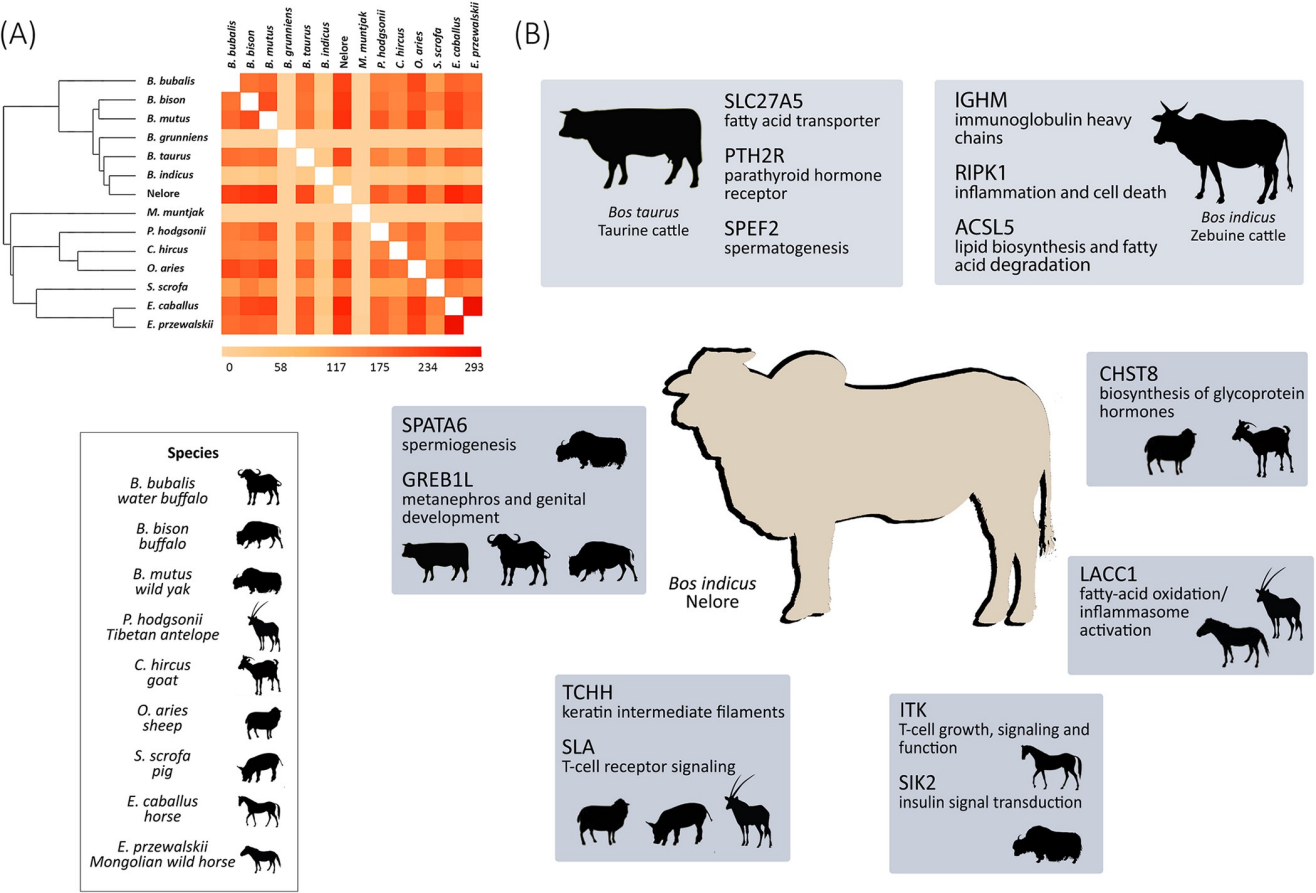
Distribution of OGs under selection and its potential functional categories. (A) Heatmap indicating the number of OGs under positive selection identified for each pair of taxa. (B) Proteins under positive selection. Each box contains one functional category and the species that presented signal of positive selection for it.

The branch-test model (ω_F_ *versus* ω_B_) was performed only on the 3,460 OGs with positive selection signal, therefore with the ones presenting negative Δω. *Muntiacus muntjak* presented the largest amount of ω > 1 (57.2%), followed by *B*. *indicus* (40.8%), and *E*. *caballus* (39.3%) (S5 Table, S4 Fig). A pairwise comparison revealed that *E*. *caballus* and *E*. *przewalskii* share the greatest amount of OGs under positive selection (293) (Fig 3A). Nelore showed a positive selection signal in 905 OGs (~39.1%), most of which were shared with *E*. *caballus* (253, ~28%) and *B*. *mutus* (244, ~27%). Some of these genes are related to insulin regulation and the immune system (Fig 3B, S6 Table). The most common annotation terms identified in regions under positive selection among all taxa were ubiquitin, tyrosine, peptidase, collagen, myosin, heat shock protein, kinase, leucine, and interleukin.

**Fig 4:**
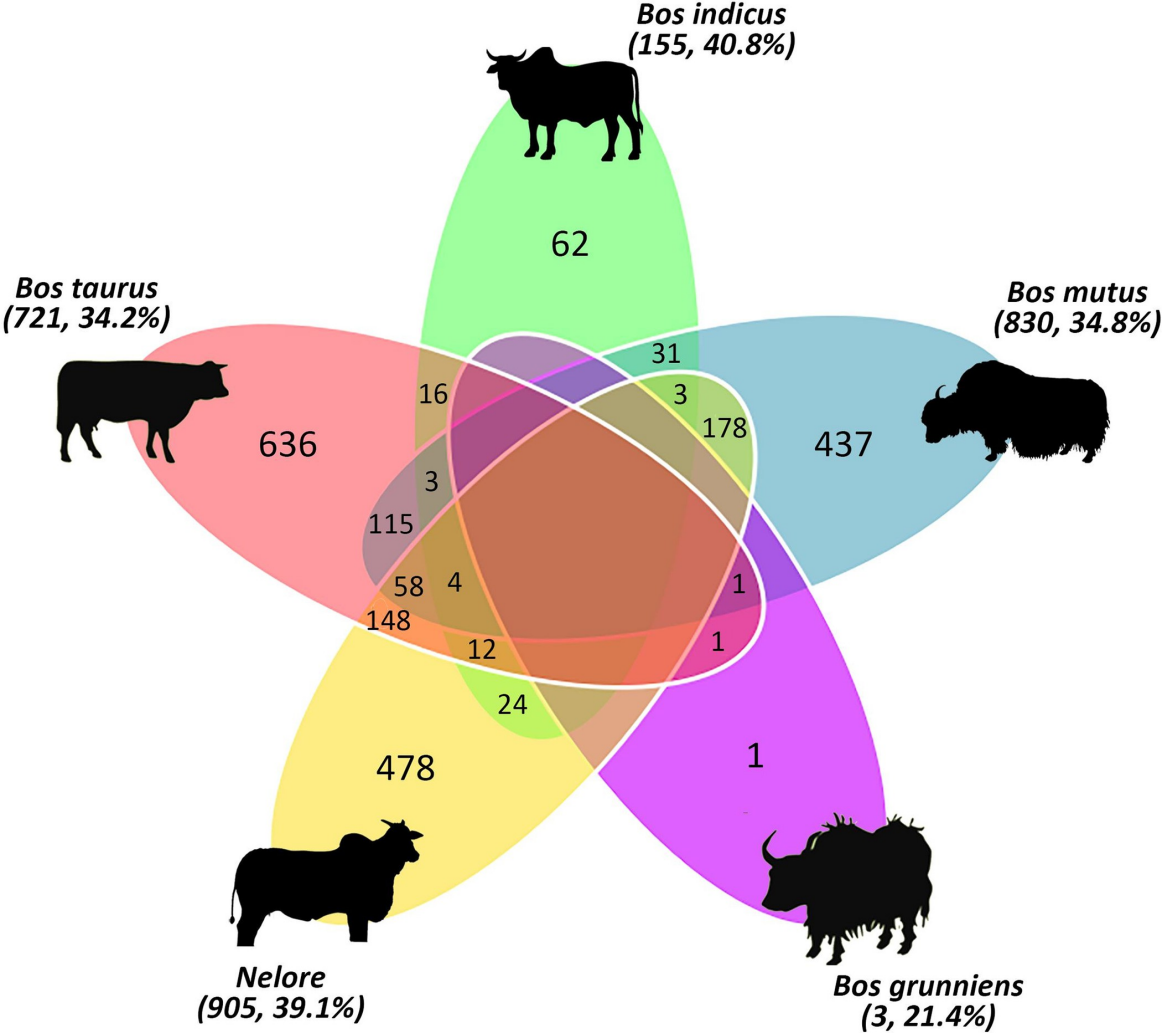
Venn diagram of OGs under positive selection in Bovinae species. Image constructed in http://www.interactivenn.net/ and obtained using multiple lists of OGs with ω > 1 per species.

Within the *Bos* genus, *B*. *indicus* (40.8%) had the highest number of OGs under positive selection, followed by Nelore (39.1%) and *B*. *mutus* (34.8%) (Fig 4). For these species, the number of OGs under positive selection shared by two or more taxa ranged from one to 293. Nelore and *B*. *taurus* shared 222 OGs under positive selection. Some of these factors are associated with parathyroid hormone, fatty acid transport, and spermatogenesis. Nelore and *B*. *indicus* presented 43 common genes, including transport protein cobalamin (*TCN2*), spermatogenesis (*SPATA2*), glycolipid metabolism (*B4GALT2*), and hormone signaling (*1JSY*) in bovines. None of the 3,460 OGs were identified as being under positive selection for all five *Bos* taxa.

### Gene family evolution

**Fig 5:**
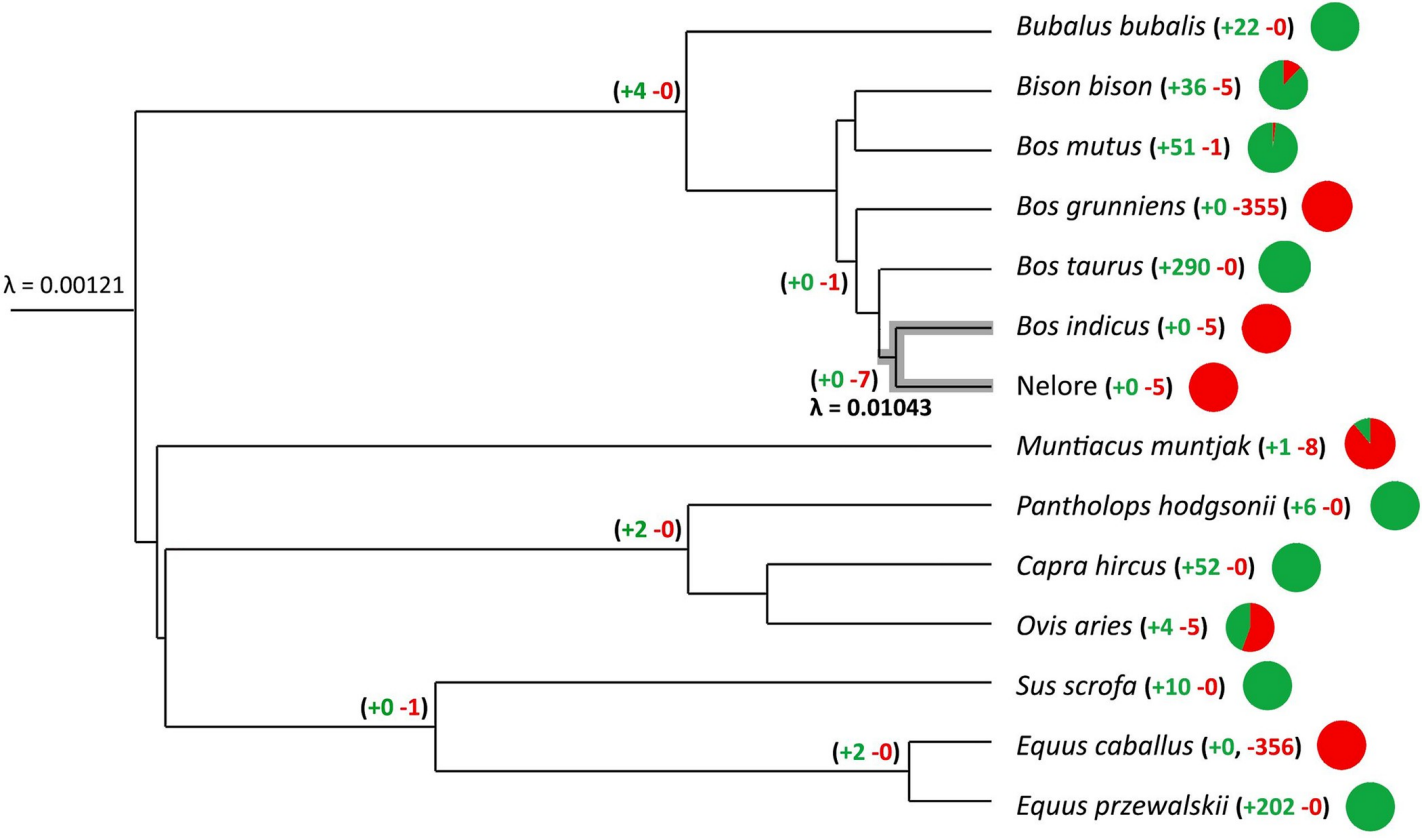
Gene family’s expansion or contraction across all branches and terminal taxa. Branch numbers indicate the number of expanding (green) and contracting gene families (red). Pie charts represent the proportion of expansions and contractions per species.

To evaluate changes in gene family size considering the phylogenetic evolutionary history in the *Bos* lineage, the CAFE software was applied. The package models gene gain and loss across lineages using a phylogenomics matrix. An IQTree phylogenomic tree was constructed from a matrix of 4,826,527 amino acid sequences, which comprised 6,850 OGs (D4). The differences in the distribution of gene families across lineages were treated appropriately by calculating the assembly error distribution (*e =* 0.80625, score = -222440.378). The assembly error distribution should not affect the obtained inferences by increasing the evolution rate along the whole tree, not reaching far beyond the implicated lineages [41]. Both the global-λ and multi-λ models were constructed assuming the error rate. According to the observed likelihood ratio, the multi-λ model fit the data better than the global model (global-λ likelihood score = -283754.196; multi-λ = -216875.694000), where the rate of gene turnover across the Nelore + *B*. *indicus* branch was almost nine times higher than that in other lineages (λ = 0.01043 *vs*. 0.00121 gains and losses/gene/million years) (Fig 5). The individual average rate of gene gain and loss for Nelore and *B*. *indicus* was identical (λ = 0.01052), only changing for the remaining lineages (λ = 0.00120 for *B*. *indicus* multi-λ model and 0.00115 for Nelore multi-λ model).

From the 13,719 OGs recovered by MCL and used in CAFE, approximately 456 (3.7%) gene families rapidly changed size in at least one lineage, including all internal branches and terminal taxa (Fig 5 for rapidly changing gene families and S5 Fig for all changes, S7 Table). To classify a change in size as rapid evolution means that the overall p-value was lower than the p-value cutoff (*p* < 0.01). The three most frequent rapidly evolving gene families appeared in eight species, which correspond to (i) the crystal structure of protocadherin from the cadherin domain, (ii) structure of the dynein complex, and (iii) structure of a bacterial cationic amino acid transporter (S8 Table). Some of the other more frequent gene families are related to the structure of the tandem SH3 domain of sorbin, crystal structure of the EphB3 kinase domain, human beta cardiac heavy meromyosin, and microtubule-associated monooxygenase, calponin, and lim domains. These proteins seem to play a role in the neural system, immune system, insulin regulation, motor function, amino acid assembly, and transmembrane transport.

Four gene families were detected in the *Bovinae* major clade, which was also the branch with the highest number of rapidly evolving gene families, all expanding in size. Their functional annotation indicated the crystal structure of pepsinogen, complex of human VARP-ANKRD1, crystal structure of RAB 6A’(Q72L), and complex of ARL2 and BART. These proteins are involved in several cellular processes, including mitochondrial transport, membrane trafficking, endocytosis, and cell differentiation [42–44], except for pepsinogen, which is an abundant protein digestive enzyme. None of the rapidly evolving families were identified in the *Bos* genus. About 9,207 gene families changing in size were detected in Nelore, but only five were classified as rapidly evolving (*p* < 0.01). These families are associated with crystal structures of pepsinogen, protocadherin gamma and bacterial cationic amino acid transporter (CAT), with structure of dynein-2 complex and EphA3 and EphA4 receptor tyrosine kinases. All of them exhibited contraction signals and encompassed 50 lost genes.

## Discussion

Due to selection to improve specific traits, domestication processes can change the morphological and/or behavioral characteristics of modern domestic animals [45]. It is not a trivial task to understand how selective pressures promote genomic changes over time, as well as identify these adaptive responses and associate them with potential phenotypic features. By using four different datasets of OGs from thirteen closely related species, we were able to obtain new insights into how the domestication process can shape these genomic changes. The differences among all species’ evolutionary histories, as well as Nelore’s advantages in tropical environments, were supported by the gene conservation analysis, which revealed genes under rapid evolution related to distinct metabolic pathways. Several features were identified in positively selected genes for multiple species, such as those related with amino acid biosynthesis, heat stress proteins, and the reproductive and innate immune system, reinforcing its relevance during the domestication process. To the best of our knowledge, the gene turnover analysis presented here has never been applied to cattle before, and revealed many lineage-specific gene losses that seem to underlie major adaptive mechanisms. Such contractions might be associated with physiological and metabolic adaptations in response to adaptive selection induced by domestication processes.

### Gene conservation

As sequence changes do not necessarily imply protein structure or function modification [46, 47], we restricted the topic discussion to slow and rapid-evolution regions that might be the result of long periods of artificial selection. This type of data can provide a reliable inference about the evolutionary rate over time due to genome responses to different environmental pressures [e.g., 48]. The sequences with low conservation (*S*_*S*_ < 0.30) were those that supposedly accumulated many changes over the generations, and in this study some of them compose GOs that play a role in fatty acid metabolism (S1 Table, S2A Fig). Fatty acid content is an important trait from a production perspective because it can affect meat softness, tenderness, and flavor [49], and is influenced by both environmental and genetic factors. Thus, this divergence might reflect the differences in meat fat content among species and their different evolutionary histories, including the domestication process [49–51]. On the other hand, many amino acid OGs with high conservation status (*S*_*S*_ > 0.70) among all taxa indicated GOs related to complex pathways, including metabolic processes, gene expression, and organelle organization (S1 Table, S2B Fig). This high conservation state might indicate an essential functional element that would remain highly conserved, most likely due to a stronger purifying selection effect, as already reported for mammals [52].

Cattle regulate their internal body temperature by sweating and breathing [3], a factor that may explain the overrepresentation of the “respiratory gaseous exchange” biological process in low conserved genomic regions (*S*_*S*_ < 0.6) between Nelore and *B*. *taurus* (S2 Table, S3B Fig). While sweating rates increase exponentially in response to higher body temperature in *B*. *indicus*, *B*. *taurus* tends to retain a greater amount of internal heat (Finch, 1986). Consequently, the more efficient thermotolerance of zebu cattle is related to reduction in growth rate [53], milk yield [54], and reproductive function [55]. In other words, evolutionary forces that led breeds to adapt to hot climates apparently resulted in the selection of genes controlling resistance to cellular heat shock [56]. Although speculative, the overrepresented GO terms related to the immune system may indicate better ectoparasite resistance in zebu cattle when compared to taurine, especially the GOs related to cytokines and T cell differentiation, which act as parasite antigens [2, 57]. Interestingly, the cytokines and T cell pathways identified here, as well as cellular activation and migration, lipid metabolism, and molecule transport, were also found by previous genome‐wide assessments and phenotype interactions studies [58–60].

Highly conserved gene clusters between Nelore and *B*. *taurus* (*S*_*S*_ = 1) included biological pathways already correlated with tenderness for *B*. *indicus* and *B*. *taurus*, myofibril assembly, positive regulation of actin cytoskeleton reorganization, and sequestering of actin monomers (S2 Table, S3A Fig) [61]. These high conservation statuses may indicate purifying selection or even introgression events, both already reported for *B*. *indicus* and *B*. *taurus* [62, 63]. An alternative but less likely hypothesis would be similar domestication processes over time, leading to efficient meat production and quality through alternative metabolic pathways in *B*. *indicus*.

### Positive selection and gene family evolution

Positive selection implies an increase in the frequency of a particular genotype/haplotype, usually caused by beneficial mutations and is commonly associated with domestication [1]. Many GO terms overrepresented within the OGs with negative ω are related to both fiber and actin assembly and steroid hormones (Fig 2, S4 Table). In bovines, steroid hormones can affect protein and fat proportions in muscles and cartilage integration [64]. Given that breeding programs are constantly selecting cattle with specific steroid interactions to improve muscle fat content, we could expect these results. Nonetheless, it is also necessary to consider domestication as a potential cause of the accumulation of deleterious mutations due to populational bottlenecks [65].

After isolating all the genomic regions under positive selection for Nelore and reanalyzing them for the 13 remaining taxa under the branch model test, we compared the variation in selective pressures across each lineage (S5 Table). Most genes revealed a neutral evolutionary rate (ω = 1) or purifying selection signal (ω < 1) (Fig 2). The variation across ω values per OG across all taxa could be explained by the particular demographic and evolutionary history of each species. Two out of the three species with the highest number of genes under positive selection are domesticated (*B*. *indicus* and *E*. *caballus*) (S4 Fig). The position of *M*. *muntjak* as the species with the highest number of genes under positive selection (57.2%), can be a bias given the lack of representative genes in the OGs (only 14 out 3,460, ~0.40%). Some of the genes under positive selection commonly found in the domesticated taxa are amino acid metabolism, cellular response, innate immune system response, heat shock proteins, collagen processes, fatty acid metabolism, hormone biosynthesis, and reproductive traits, highlighting the relevance of metabolic pathways when considering the domestication process (Fig 3, S6 Table).

Considering the high number of gene families analyzed, only 456 (~3.7%) were classified as rapidly changing. Several functional categories could be identified among these rapidly evolving gene families, including histone binding, immune defense/stress, cell signaling, and reproduction. The last three have already been reported in a previous study on mammals [14]. Furthermore, some of these functions were also present in the most abundant annotation terms in genes under positive selection (Fig 3), such as myosin related, heat shock protein, leucine, kinase protein, tyrosine, and ubiquitin, reinforcing the selective pressure on these traits. Both Nelore and *B*. *taurus* presented at least 34% of the genes under positive selection. Because the transcriptome of Nelore was entirely obtained from the *longissimus dorsi* muscle, an increase in overrepresented GOs related to carbohydrate and lipid metabolism could be expected, as reported by Poleti *et al*. [19]. Here, we identified genes associated with glycolisis (GAPDHS, FBP2, ALDOC), fatty acid degradation/biosynthesis (ELOVL7, SCD, DECR2, CRYL1), and amino acid degradation (ALDH1A1, CRYL1, ABAT, NUDT13, CTPS2, NME4, and ENTPD3). Some other protein families previously associated with lipogenesis and/or adipogenesis were also identified, such as HSPs (HSP40, HSPA4, HSPA8, and HSP110), E3 ubiquitin-protein ligase (*RNF213*), and sterol regulatory element-binding proteins (*SREBPs*), found in cattle species with high intramuscular fat content [66]. Even using this distinct approach, we were able to find many genes under positive selection overlapping with both Somavilla *et al*. [9] and Randhawa *et al*. [67] meta-assembly results, providing robustness to these results. Their functional roles included purine and thiamine metabolism (ZRANB3, KIF1B), transglutaminase 1 synthesis (TGM1), thyroglobulin function (TG), fibroblast growth factors (FGF10, FGF11, TGFB3), protocadherins (PCDH12, PCDH9, PCDH17, PCDH16), stem cell factor (SCF), and reproductive traits.

The small difference between the average λ obtained for the multi-λ model individually performed for Nelore and *B*. *indicus* reinforces that both lineages show equivalent gene losses rate, regardless of the type of data used (transcriptome and genome sequences). The gene families rapidly expanding in bovine lineages play different roles in cellular processes, including organelle transport, endocytosis, cell growth, and differentiation (S8 Table). Expansion events can be indicative of adaptive selection in phenotypically relevant genes [68, 69]. For this reason, the presence of the pepsinogen, a protein digestive enzyme, could be particularly interesting for future animal breeding studies due to its use as an indicator of gastric infections by nematodes in ruminants [70].

From the five gene families in contraction in Nelore, metabolic pathways associated with amino acid transport, immune system, nervous system, digestive tract, and motor transport were identified. These gene families seem to play important roles in specifying cell–cell connections in mammalian brains, resistance to parasites in domesticated species (protocadherins [71]), cellular transport and motor function (dynein complexes [72]), cell–cell interactions/topographic ordered connections at the visual system (Eph receptors [73, 74]), cationic amino acid transporters/macrophage activation [75] and proteolytic activity in digestive tract (pepsinogen). The loss of whole gene families has been reported for several taxa [e.g., 17, 16], and is frequently associated due to changes in environmental conditions that lead to morphological, physiological, and metabolic adaptations [17]. Lineage-specific gene losses might underlie major adaptive mechanisms through the rapid evolution of protein sequences until they are no longer identified as belonging to the same family [68]. Therefore, we could expect that these contractions would be produced as an adaptive natural selection response, most likely as a sub-product of domestication processes.

Our study evaluated the evolutionary history and selection signatures in Nelore and other closely related domesticated taxa, including *Bos* species, such as *B*. *taurus*. Data showed that domesticated species presented the highest density of genes under positive selection, as expected. Several features with substantial relevance from a domestication perspective, including cellular response to heat stress, amino acid biosynthesis, and immune system-related, were identified in two or more of our analyses, many of which have already been reported in previous studies, supporting robust data and selective pressures in these traits over time. The functional categories from these gene clusters agree with many of the known advantages that Nelore has when compared to taurine cattle in tropical environments. Our data not only reinforce which phenotypic traits are most affected by selective pressures, but also reveal how the genome changes may respond to different evolutionary histories, especially under intense domestication. For instance, the genes described as under positive selection in Nelore are candidates to contribute to the higher immune resistance and more efficient heat dissipation seen in this breed and could be considered as targets for genetic improvement in cattle. Thus, by providing a more specific understanding of genomic responses to artificial selection, this work may lay out a basis for future animal genetic improvement investments.

## Supporting information

S1 Fig Similarity score distribution for multiple taxa comparisons. Similarity score (A) among all analyzed taxa, and (B) between Nelore and *Bos taurus* amino acid orthologous groups. The y-axis represents the density and the x-axis, the similarity score from zero to one (identical sequences).
(TIF)

S2 Fig Enriched Gene Ontology (GO) network with the overrepresented GOs from orthologous groups containing all taxa. Overrepresented GOs from orthologous groups with similarity score (A > 0.70 and (B) < 0.30 among species. Each circle represents one single over-represented GO term and the solid lines indicates the biological connection between them. The colors indicate the different metabolic categories. The discussed categories are indicated with black borders.
(TIF)

S3 Fig Enriched Gene Ontology (GO) network with the overrepresented GOs from orthologous groups containing Nelore and *Bos taurus*. Overrepresented GOs from orthologous groups with similarity score (A) = 1 and (B) < 0.60 between Nelore and *Bos taurus*. Each circle represents one single over-represented GO term and the solid lines indicates the biological connection between them. The colors indicate the different metabolic categories. The discussed categories are indicated with black borders.
(TIF)

S4 Fig Branch-test results for 3,460 orthologous groups (OGs). The amount of OGs with ω > 1 (y-axis) are shown per species (x-axis). The black line indicates only the number of OGs with ω > 1, while the dashed line represents the total number of OGs which the species is present (occupancy).
(TIF)

S5 Fig Distribution of changes in gene family size. The number of gene gains (in green) and losses (in red) is provided per branch/terminal taxa.
(TIF)

S1 Table Enrichment Gene Ontology (GO) results for amino acid orthologous group with extreme low and high similarity score among all taxa. For each GO term, the similarity score, GO ID, name and category, attached p-value (p < 0.05), the number of sequences that are annotated with the GO in the test-set and reference, are shown.
(XLSX)

S2 Table Enrichment Gene Ontology (GO) results for amino acid orthogroups with extreme low and high similarity score between Nelore and *Bos taurus*. For each GO term, the similarity score, GO ID, name and category, attached p-value (p < 0.05), the number of sequences that are annotated with the GO in the test-set and reference, are shown.
(XLSX)

S3 Table ETE Toolkit results for nucleotide orthologous groups (OGs) under models M1 and M2. The obtained values are shown for each OG alongside with its p-value. The p-values < 0.05 are indicated with asterisks.
(XLSX)

S4 Table Enrichment Gene Ontology (GO) results for orthologous groups with negative Δω. For each GO term, the similarity score, GO ID, name and category, attached p-value (p < 0.05), the number of sequences that are annotated with the GO in the test-set and reference, are shown.
(XLSX)

S5 Table Branch-site results of 3,460 orthologous groups (OGs) per species. Each line represents an OG, and the values attached indicated the respective dN/dS ratio (ω) or the lack of sequence (“NA”) per species.
(XLSX)

S6 Table Annotation results for 3,460 orthologous groups (OGs) presenting negative Δω. The table contains, for each OG, the Gene Ontology (GO) identified with blastp tool, the e-value obtained from the search, the GO description, ID and names.
(XLSX)

S7 Table Gene family turnover analysis results for all branches and terminal taxa. Each line contains the results of each branch or terminal taxa. The columns indicate, respectively, the number of expanding gene families, genes gained, contracting gene families, lost genes, gene family that did not presented changes, the average rate of expansion, the number of rapidly evolving families and, finally, the ID of rapidly evolving families. The values within parenthesis indicate the number of rapidly expansions/contractions, while the values within brackets indicated the number of genes gained (+) and lost (-).
(XLSX)

S8 Table Annotation results for 456 rapidly evolving gene families. For each gene family, the table shows the terminal taxa presenting rapidly evolution, the average length, the subject sequence ID, the e-value and the description attached to the blastp search.
(XLSX)
