# Supplementary figures and images for: Molecular evolution and signatures of selective pressures on Bos, focusing on the Nelore breed (Bos indicus)
Source: PLoS One. 2022 Dec 22;17(12):e0279091. doi: 10.1371/journal.pone.0279091 (PMC9778527; doi:10.1371/journal.pone.0279091)

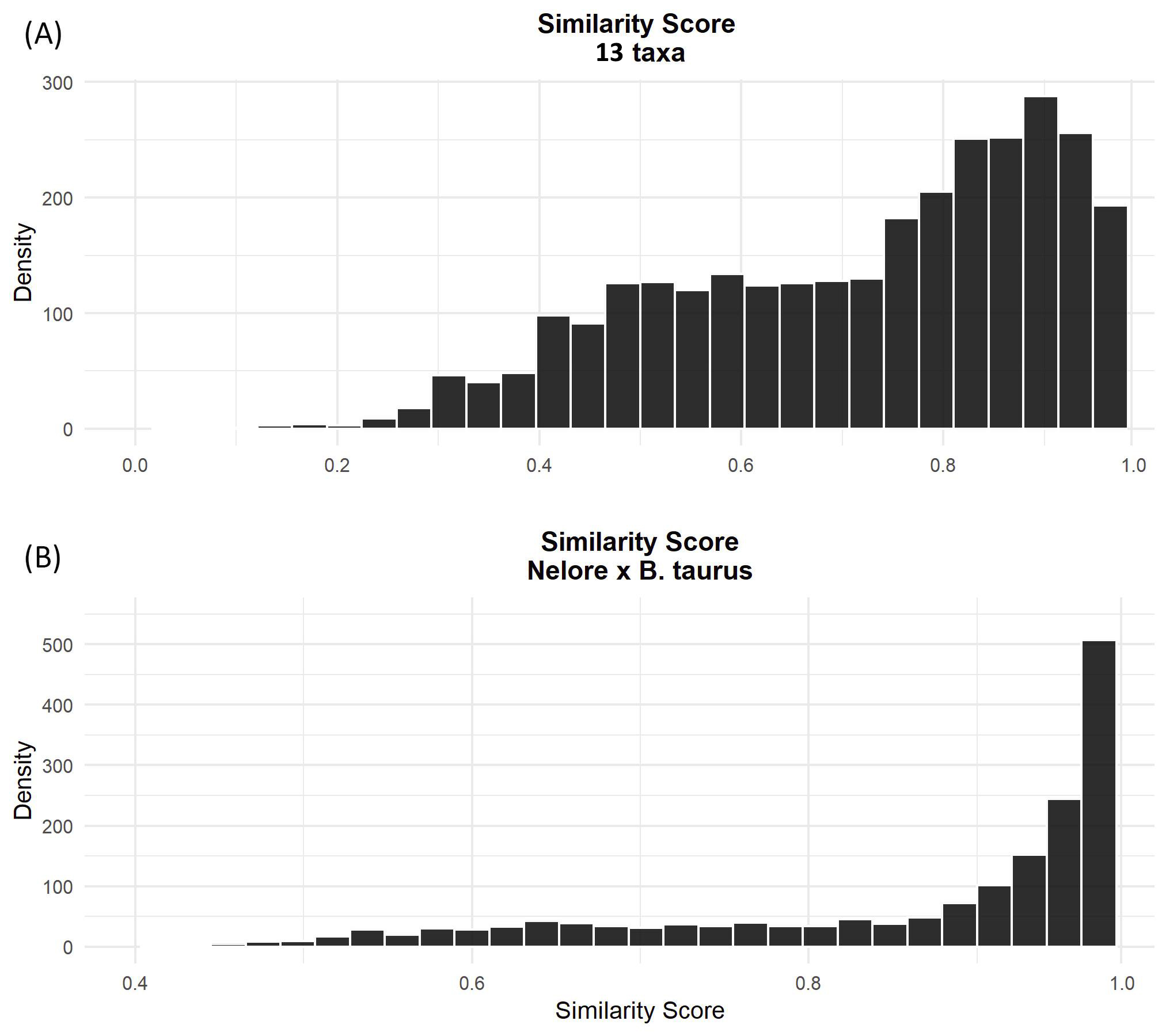

Supplement: S1 Fig — Similarity score (A) among all analyzed taxa, and (B) between Nelore and Bos taurus amino acid orthologous groups. The y-axis represents the density and the x-axis, the similarity score from zero to one (identical sequences). (TIF) [file pone.0279091.s001.tif]

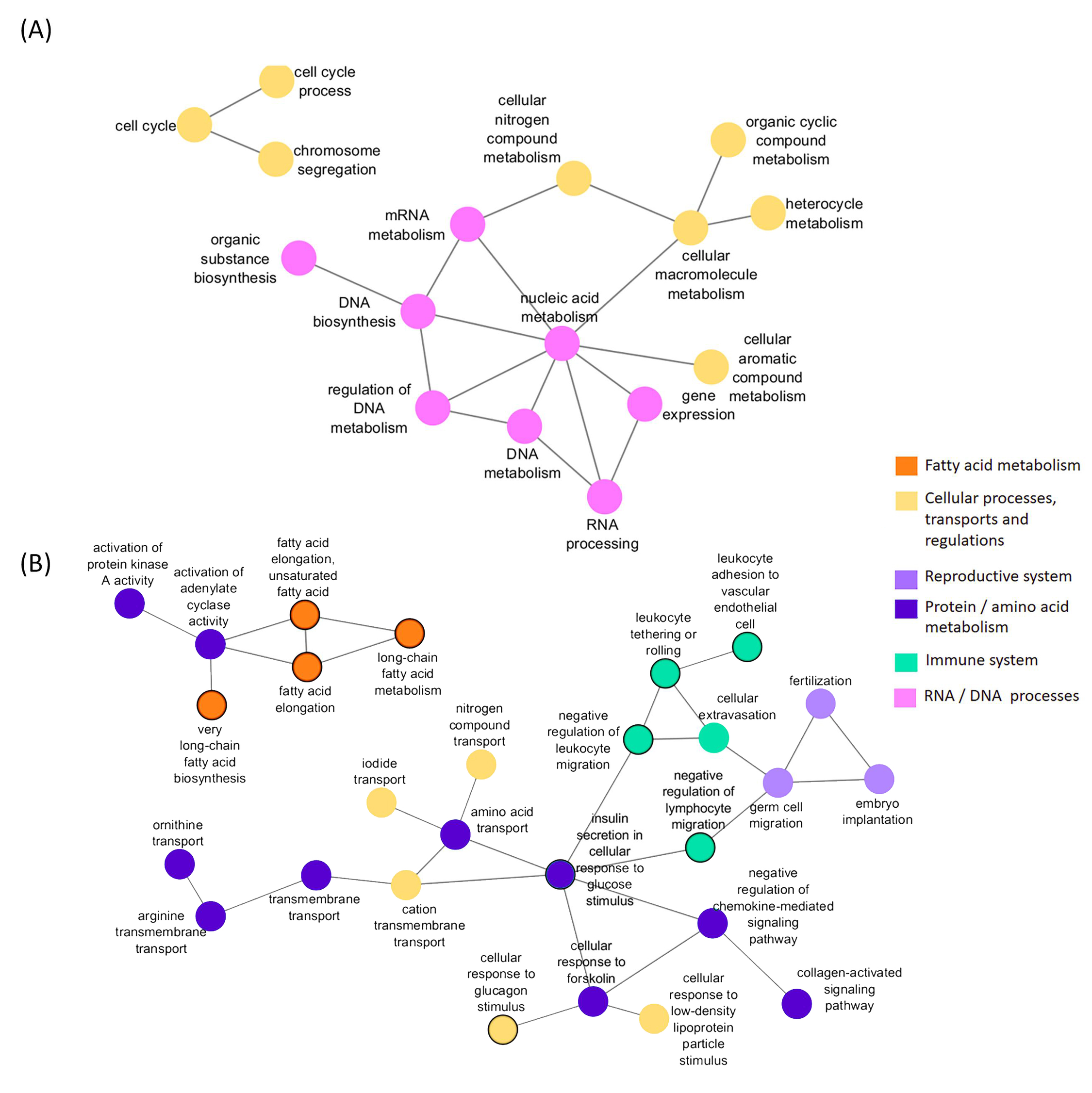

Supplement: S2 Fig — Overrepresented GOs from orthologous groups with similarity score (A > 0.70 and (B) < 0.30 among species. Each circle represents one single over-represented GO term and the solid lines indicates the biological connection between them. The colors indicate the different metabolic categories. The discussed categories are indicated with black borders. (TIF) [file pone.0279091.s002.tif]

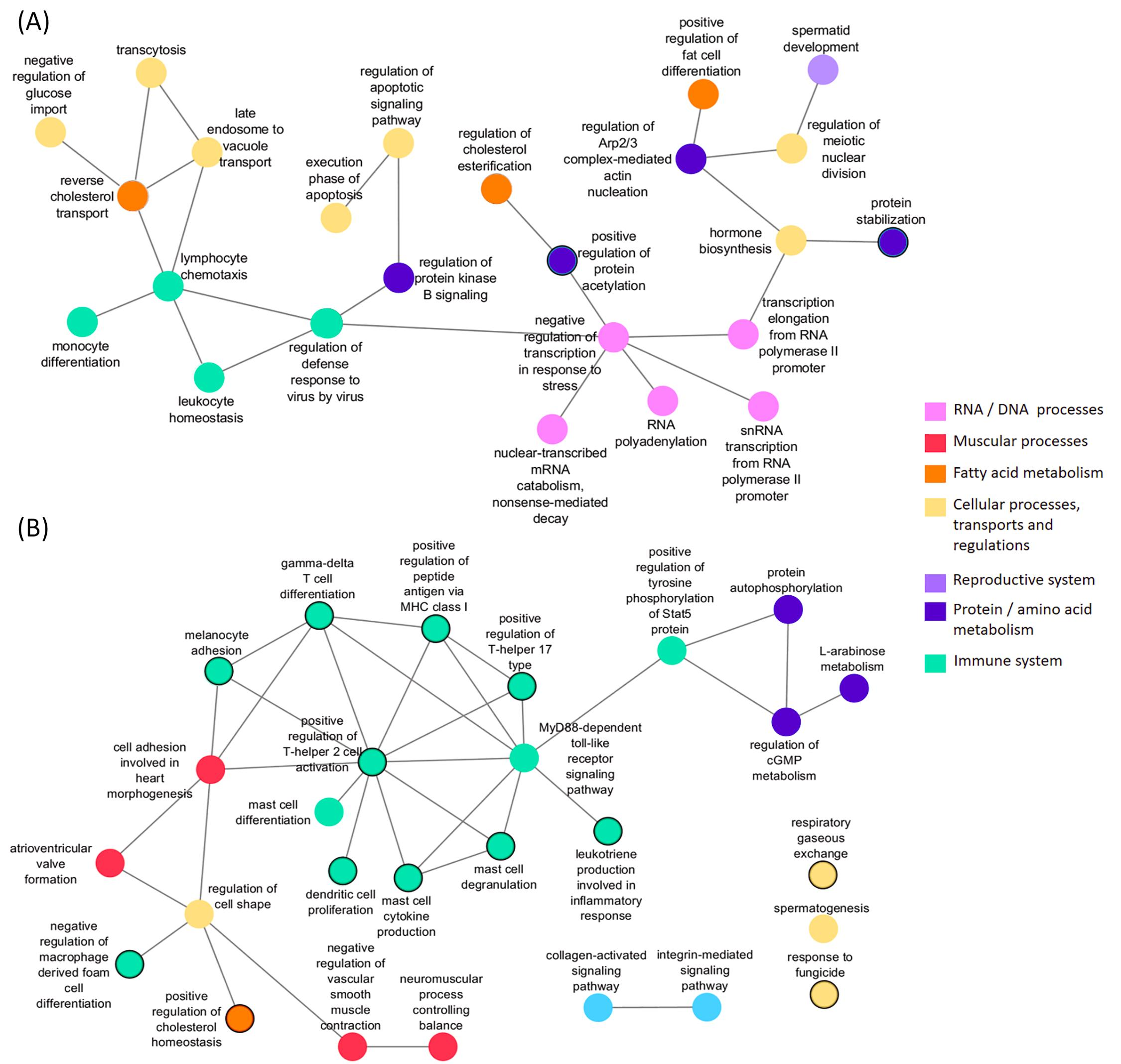

Supplement: S3 Fig — Overrepresented GOs from orthologous groups with similarity score (A) = 1 and (B) < 0.60 between Nelore and Bos taurus. Each circle represents one single over-represented GO term and the solid lines indicates the biological connection between them. The colors indicate the different metabolic categories. The discussed categories are indicated with black borders. (TIF) [file pone.0279091.s003.tif]

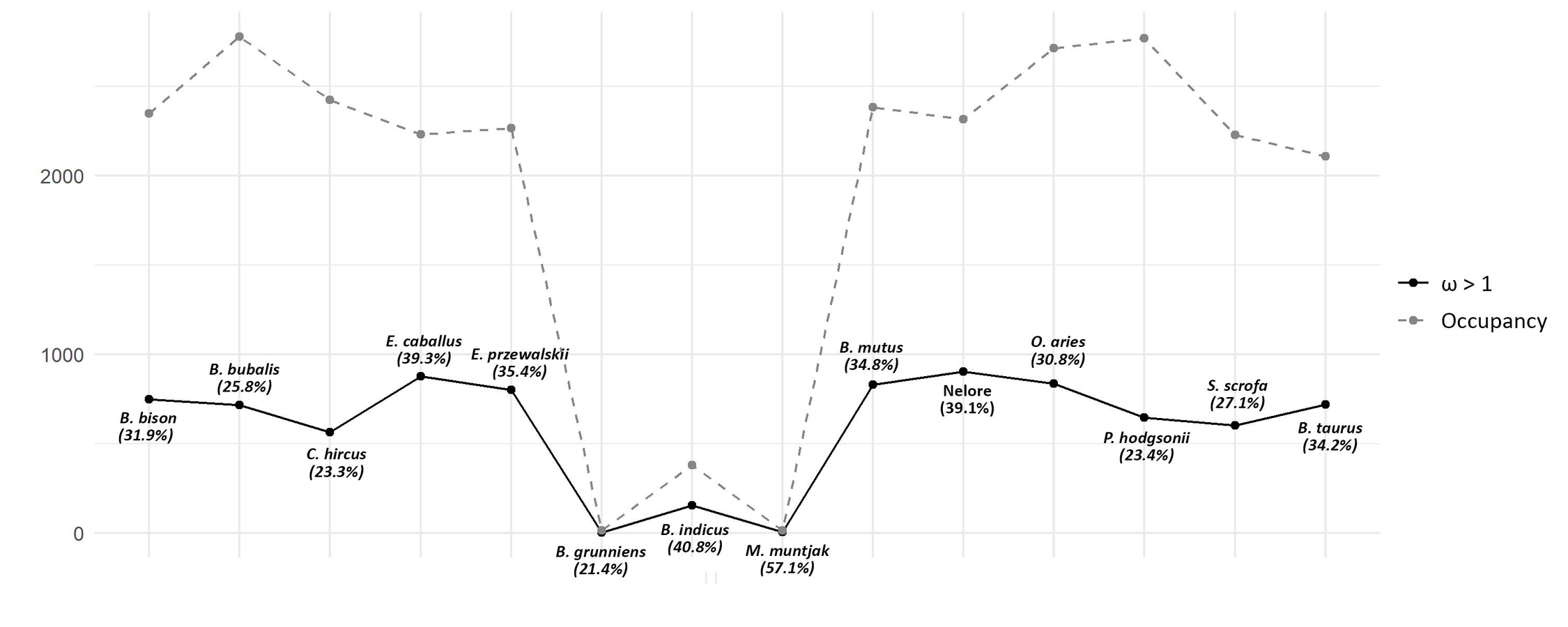

Supplement: S4 Fig — The amount of OGs with ω > 1 (y-axis) are shown per species (x-axis). The black line indicates only the number of OGs with ω > 1, while the dashed line represents the total number of OGs which the species is present (occupancy). (TIF) [file pone.0279091.s004.tif]

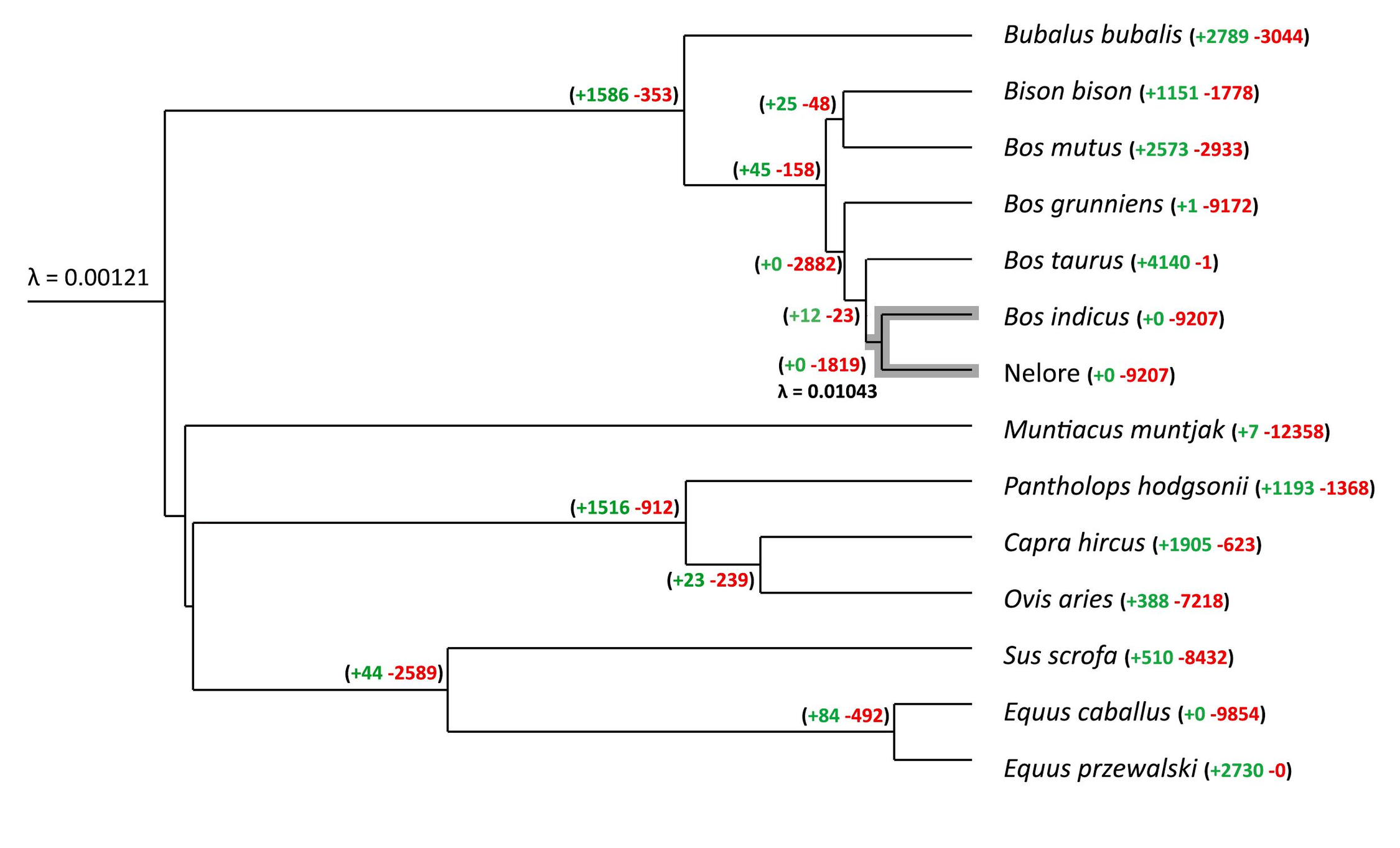

Supplement: S5 Fig — The number of gene gains (in green) and losses (in red) is provided per branch/terminal taxa. (TIF) [file pone.0279091.s005.tif]
